# Drug‐eluting bead chemoembolization for the treatment of nonresectable hepatic carcinoma in dogs: A prospective clinical trial

**DOI:** 10.1111/jvim.16109

**Published:** 2021-05-06

**Authors:** Cleo P. Rogatko, Chick Weisse, Tobias Schwarz, Allyson C. Berent, Marcio A. Diniz

**Affiliations:** ^1^ The Animal Medical Center New York New York USA; ^2^ Veterinary Surgical Centers Vienna Virginia USA; ^3^ Royal (Dick) School of Veterinary Studies The University of Edinburgh Roslin United Kingdom; ^4^ Cedars‐Sinai Medical Center Los Angeles California USA

**Keywords:** DEB, doxorubicin, interventional radiology, liver, TACE

## Abstract

**Background:**

Effective treatment options for nonresectable hepatic carcinoma (HC) in dogs are limited.

**Hypothesis/Objective:**

Objectives were to report outcomes, complications, and tumor responses via computed tomography (CT) assessment after drug‐eluting bead transarterial chemoembolization (DEB‐TACE) for nonresectable HC in dogs. The authors hypothesized that major complications would be uncommon and short‐term CT assessment would demonstrate stable disease or partial response.

**Animals:**

Client‐owned dogs (n = 16) with nonresectable HC.

**Methods:**

Prospective, single‐arm clinical trial. Drug‐eluting bead transarterial chemoembolization was performed to varying levels of blood flow stasis. Computed tomography imaging was compared before and approximately 12 weeks after initial treatment.

**Results:**

Drug‐eluting bead transarterial chemoembolization was successfully administered in all attempts. Based on percent change in elliptical tumor volume response (mL), stable disease (8/13; 62%) was the most common outcome followed by partial response (3/13; 23%) and progressive disease (2/13; 15%) with a median of 74 days (range, 39‐125) after initial treatment. Median tumor volume (mL) after DEB‐TACE decreased in volume by 13% (range, 56% decrease to 77% increase). Mild complications consistent with postembolization syndrome occurred after 7/27 (26%) treatments. Major complications occurred after 3/27 (11%) treatments: hepatic abscess/septicemia (2) and cholecystitis/death (1), resulting in treatment‐induced death after 2/27 (7%) treatments. Median survival time after treatment was 337 days (range, 22‐1061). Dogs with a presenting complaint of weight loss (*P* = .02) had a significantly shorter median survival time (126 days; range, 46‐337) than those dogs without prior history of weight loss (582 days; range, 22‐1061).

**Conclusions:**

Drug‐eluting bead transarterial chemoembolization for nonresectable HC is a feasible procedure, which promoted stable disease or partial response in 85% of dogs in this study sample.

**Abbreviations**
CT, computed tomography; CTA: computed tomography angiography
DEB‐TACE: drug‐eluting bead transarterial chemoembolization
HC: hepatic carcinoma
HCC: hepatocellular carcinoma
MST: median survival time
PES: post‐embolization syndrome
RECIST: Response Evaluation Criteria in Solid Tumors
TACE: transarterial chemoembolization
TAE: transarterial embolization

## 1 INTRODUCTION

Hepatic carcinomas (HCs) are the most common primary liver neoplasm in dogs with hepatocellular carcinoma (HCC) comprising the most common subset,^1^, ^2^, ^3^ followed by bile duct carcinomas,^2^, ^4^, ^5^, ^6^ and hepatic neuroendocrine carcinomas.^4^, ^7^, ^8^ For massive, resectable HCs, complete surgical resection is recommended.^3^, ^9^, ^10^ The prognosis for dogs with focal, resectable HC is good with surgical intervention (range, >1460 to >1836 days)^3^, ^9^, ^10^, ^11^; however, there is minimal information regarding the efficacy of nonsurgical treatment modalities for nonresectable disease.

In humans, transarterial embolization (TAE) or transarterial chemoembolization (TACE) are important components of treatment for nonresectable HC.^12^, ^13^, ^14^, ^15^, ^16^, ^17^ TAE involves transcatheter superselective arterial injections of embolic agents into the tumor‐feeding arteries resulting in targeted ischemic necrosis.^18^, ^19^ Drug‐eluting bead TACE (DEB‐TACE) delivers higher doses of chemotherapeutic agents with longer drug‐tumor contact times by administering embolic beads which slowly elute chemotherapeutics locally, reducing systemic adverse effects.^19^ In people with nonresectable HC, TACE shows improved median survival time (MST) vs conservative management.^20^, ^21^, ^22^, ^23^, ^24^ Compared to conventional TACE, DEB‐TACE might show comparable or improved efficacy and fewer adverse effects,^25^, ^26^, ^27^, ^28^, ^29^, ^30^ leading to the current investigation of DEB‐TACE in dogs.

The objectives of this study were to report outcomes, complications, and computed tomography (CT) elliptical tumor volume response rates associated with administration of 100 to 300 μm doxorubicin DEB‐TACE to dogs with nonresectable HC. The authors hypothesized that major^31^ complications associated with DEB‐TACE treatment would be uncommon and that diagnostic imaging would demonstrate short‐term stable disease (SD) or partial response after DEB‐TACE.

## 2 MATERIALS AND METHODS

### 2.1 Case selection

Dogs diagnosed with nonresectable HC, either naïve tumors or those with nonresectable regrowth after previous excision at The Animal Medical Center, New York from April 2010 to July 2015 were prospectively enrolled in the clinical trial and treated with DEB‐TACE after informed owner consent. The study was approved by the Institutional Animal Care and Use Committee.

Dogs were included if a cytologic or histologic diagnosis of HC was obtained and if the mass was determined by a surgeon to be nonresectable without substantial risk when assessed by either preoperative computed tomography angiography (CTA) or during exploratory laparotomy. Dogs were excluded if they were treated with chemotherapy, radiation therapy, or surgical intervention other than surgical biopsy within the previous 3 months.

Dogs were staged with standard techniques including physical examination, 3‐view thoracic radiography or thoracic CT scan, abdominal CTA, complete blood cell count, serum biochemistry profile, resting bile acids, and urinalysis. The first treatment was performed within 30 days of staging.

### 2.2 Medical records review

Medical records review was performed, and data recorded included signalment (age, breed, and sex), weight, presenting clinical signs at diagnosis of HC, prior surgeries, biopsy and cytologic findings, history of abdominal effusion, diagnostic imaging findings and tumor volume measurements, DEB‐TACE procedural information and schedule, duration of procedure, procedural complications, postprocedural complications, survival times (STs), and necropsy results.

### 2.3 Treatment protocol

Dogs were to receive 2 DEB‐TACE procedures 6 weeks apart. The goal of the initial DEB‐TACE procedure was prolonged chemotherapeutic drug‐delivery through administration of drug‐eluting beads within the tumor while preserving blood flow through the lobar and first‐order hepatic arteries, enabling subsequent treatments. The goal of the second treatment was to repeat chemotherapeutic drug‐delivery and then additionally provide embolization to vascular stasis, defined as no evidence of continued hepatic arterial flow to the tumor or lack of tumor blush.^32^At the conclusion of the second treatment, if vascular stasis had not yet occurred, additional nondrug loaded beads (LC/DC Bead 100‐300 μm hydrogel microspheres, Biocompatibles UK Limited, Farnham, UK) were administered until vascular stasis was achieved.

The DEB‐TACE procedure was performed as described previously^33^, ^34^, ^35^ under general anesthesia via a femoral artery or carotid artery approach (Micropuncture set, Infiniti Medical, Menlo Park, California). After superselective hepatic arterial branch access to the main arterial supply of the mass, 100 to 300 μm DEBs (LC/DC Bead 100‐300 μm hydrogel microspheres, Biocompatibles UK Limited) loaded with 30 mg/m^2^ of doxorubicin (or 1 mg/kg if under 10 kg) (Adriamycin, doxorubicin hydrochloride, Pfizer Inc, Andover, Massachusetts) was administered. Angiography was performed after each DEB‐TACE procedure to determine whether vascular stasis had been achieved.

Dogs were discharged the day after the procedure and prescribed omeprazole (Prilosec, Proctor and Gamble, Cincinnati, Ohio) (1 mg/kg PO q12h for 7‐14 days), ampicillin/clavulanate (Clavamox, Pfizer Animal Health, Madison, New Jersey) (13.75 mg/kg PO q12h for 14 days), ondansetron (Zofran, GlaxoSmithKline, Philadelphia, Pennsylvania) (0.1‐1 mg/kg PO q24h for 7 days), and prednisone (Prednisone, Roxane Laboratories Inc, Columbus, Ohio) (1 mg/kg/d PO × 3 days, then 0.5 mg/kg/d PO × 3 days, then 0.5 mg/kg/d q48h for 3 doses.^35^

### 2.4 Tumor response evaluation

All dogs had baseline multiphase (arterial with multiple venous phase) abdominal CT angiography (CTA) performed within 2 days before initial DEB‐TACE. Six weeks later, a second DEB‐TACE treatment was performed. After each treatment, a noncontrast abdominal CT was performed under the same anesthetic even. A final multiphase CTA was performed ~12 weeks after the first treatment in conjunction with doxorubicin (Adriamycin, doxorubicin hydrochloride, Pfizer Inc, Andover, Massachusetts) administered IV (as part of a separate pharmacokinetics study) (30 mg/m^2^ IV once, or 1 mg/kg if a dog weighed less than 10 kg). Three dogs (dogs 2, 7, and 11) did not have a follow‐up CTA performed after DEB‐TACE; tumor response could not be assessed for these dogs.

CTA image series were reviewed by a board‐certified radiologist (T. S.) and procedures performed using a dedicated workstation (Syngo.via, Siemens AG, Erlangen, Germany) and oncological imaging software (Syngo.MM Oncology, Siemens AG, Erlangen, Germany). CTA's pre‐ and post‐treatment were compared for the same individual. Subjective assessment was performed for hepatic lesion description (number of lesions and lobes involved). Manual correction of hepatic lesion borders was performed after automatic delineation (Syngo.via, Siemens AG; Syngo.MM Oncology, Siemens AG). Response to treatment was generated by the system in tumor volume (in mL).^36^, ^37^ Elliptical tumor response was categorized as complete response: complete resolution of all disease; partial response: ≥30% decrease size; SD: percent difference in size between −30% and 20%; and progressive disease ≥ 20% increase in size.^38^, ^39^, ^40^

### 2.5 Complications

At each visit, a detailed history was obtained from the owner. Complications attributable to the procedure were considered mild if able to be managed by either outpatient or inpatient interventions that did not require anesthesia, or major if requiring urgent intervention under anesthesia for resolution or resulting in death.^31^

### 2.6 Statistical analysis

Survival time was defined as the duration from the date of DEB‐TACE treatment to the time of death. Descriptive measures were presented as median and range or quantitative variables and frequency (percentage) for qualitative variables. Independent groups were compared using Fisher for qualitative variables, and *t* test, Mann‐Whitney or Brunner‐Munzel test for quantitative variables based on the assumption of normality and equality of variances. Homoscedasticity was tested using Levene test, and normality was tested using Shapiro‐Francia test. Survival curves were presented using Kaplan‐Meier estimator, and simple proportional hazard Cox models were fitted to estimate hazard ratios with 95% confidence intervals. Marginal homogeneity was tested using McNemar test. Agreement was classified based on the Kappa statistic based on 0 to 0.2 no agreement, 0.21 to 0.40 slight agreement, 0.41 to 0.60 moderate agreement, 0.61 to 0.80 substantial agreement, 0.81 to 1 almost perfect agreement.^41^ All hypotheses were 2‐sided at 5% significance level. Calculations were performed using R‐package, version 3.6.1 (R Core Team [2019]. R: A language and environment for statistical computing. R Foundation for Statistical Computing, Version 3.6.1, Vienna, Austria. https://www.R-project.org/).

## 3 RESULTS

Sixteen dogs diagnosed with nonresectable HC, either naïve tumors (12/16; 75%) or those with suspected regrowth after previous excision (4/16; 25%) and treated with DEB‐TACE at The Animal Medical Center satisfied study inclusion criteria. There were 8 male castrated dogs, 7 female spayed dogs, and 1 intact male. The median age at the time of first treatment was 11.1 years (range, 5.8‐13.2 years). The median weight was 14.7 kg (range, 6.3‐30.8 kg). Breeds represented included Shih Tzu (3), Labrador retriever (1), German wirehaired pointer (1), Schnauzer (1), Beagle (1), Australian Shepherd (1), Pekingese (1), and mixed breed (7). Presenting complaints at the time of HC diagnosis and pertinent history are shown in Table 1. The diagnosis of a high risk or nonresectable hepatic mass was determined via exploratory laparotomy in 10/16 dogs (62%) at referral surgical institutions and via CT in 6/16 dogs (38%). The diagnosis of HCC was determined via laparoscopic biopsy, ultrasound‐guided needle core biopsy or surgical biopsy in 15 dogs (15/16 dogs; 94%) and via fine‐needle aspirate cytology in 1 dog (1/16; 6%).

**TABLE 1:** Associations of historical signs and presenting complaint with survival via univariable cox regression for dogs with HC after DEB‐TACE

Variable	Count (%)	Estimate (95% confidence interval)	*P* value
History of previous hepatic mass resection		0.509 (0.157‐1.65)	.26
No	12 (75)		
Yes	4 (25)		
History of hemoabdomen		0.480 (0.110‐2.14)	.33
No	13 (81.3)		
Yes	3 (18.8)		
Presenting complaint of inappetence		1.51 (0.522‐4.36)	.45
No	10 (62.5)		
Yes	6 (37.5)		
Presenting complaint of weight loss		4.82 (1.23‐18.9)	**.02** ^*^
No	11 (68.8)		
Yes	5 (31.3)		
Presenting complaint of lethargy		3.16 (0.695‐14.4)	.14
No	13 (81.3)		
Yes	3 (18.8)		
Presenting complaint of vomiting		2.59 (0.724‐9.29)	.14
No	11 (68.8)		
Yes	5 (31.3)		
Presenting complaint of diarrhea		2.64 (0.626‐11.1)	.19
No	13 (81.3)		
Yes	3 (18.8)		
Presenting for incidental blood work finding		0.906 (0.280‐2.94)	.87
No	12 (75)		
Yes	4 (25)		
Presence of concurrent neoplasia		0.732 (0.257‐2.09)	.56
No	8 (50)		
Yes	8 (50)		

Abbreviations: DEB‐TACE, drug‐eluting bead transarterial chemoembolization; HC, hepatic carcinoma.

^*^ Bold values are statistical significance *P* < .05.

All dogs had an abdominal CTA performed 0 to 1 days before treatment. On initial CTA, 12/16 (75%) had masses solely or including the right side of the liver while 2/16 (12.5%) were solely left and 2/16 (12.5%) were centrally located masses.

Initial DEB delivery was technically successful in all dogs, meaning that vascular access was achieved, the major feeding artery selected, and the DEBs (LC/DC Bead 100‐300 μm hydrogel microspheres, Biocompatibles UK Limited) delivered. Fourteen out of 16 dogs (88%) initially received a full 30 mg/m^2^ doxorubicin dose (Adriamycin, doxorubicin hydrochloride, Pfizer Inc). Two dogs (dog 10 and 11) received 89% and 75% of the dose, respectively, before stasis was achieved, preventing additional bead delivery. Four out of 16 dogs (25%) were embolized to stasis at initial treatment. The median total procedural duration was 85 minutes (range; 55‐134).

A second DEB‐TACE treatment was attempted in 11/15 (73%) dogs; 1 dog (dog 2) developed fatal complications after the first DEB‐TACE (see Section 3.2). Two dogs had been embolized to stasis before to receiving the full treatment volume and identifying an accessible vascular supply to the tumor was considered unlikely. It was not clear from the records why a second procedure was not pursued in 2 dogs. All 11 dogs successfully received a second DEB‐TACE treatment to vascular stasis on the first attempt. Due to previous embolization to stasis, 1 dog received 15% of the full treatment volume and stasis was complete; the 10 remaining dogs embolized to stasis received a full treatment volume. Four dogs had an additional 0.25 mL of DEBs (without chemotherapy added) (LC/DC Bead 100‐300 μm hydrogel microspheres, Biocompatibles UK Limited) administered after administration of the initial dose to achieve tumor stasis (dogs 3, 8, 9, 14). The median total procedural duration was 91 minutes (range, 70‐125).

### 3.1 Chemotherapy administered IV

Nine out of 13 (69%) remaining dogs received a single intravenous doxorubicin (Adriamycin, doxorubicin hydrochloride, Pfizer Inc) treatment (30 mg/m^2^) at the time of second CTA. Three dogs did not survive to repeat CTA. One dog developed complications from a hepatic abscess and did not undergo repeat CT/chemotherapy. It was unclear from the record why 2 dogs did not receive chemotherapy. One dog developed systemic mastocytosis 1 month after DEB‐TACE treatments and received intravenous treatments of vinblastine and toceranib phosphate over the next 2 months (dose regimen unknown) after second CTA. Eleven (11/16; 69%) dogs received both DEB‐TACE treatments and 8/16 (50%) dogs received all 3 treatments (Two DEB‐TACE plus IV doxorubicin [Adriamycin, doxorubicin hydrochloride, Pfizer Inc] chemotherapy).

See Table 2 for tumor volume measurements before and after embolization.

**TABLE 2:** Computed tomography tumor volume measurements before and after DEB‐TACE for dogs with HC as risk factors for survival

CT tumor size measurement	Median (range)	*P* value	Median (range)	*P* value	Median (range)	*P* value
	Before DEB‐TACE		After DEB‐TACE		Percent change following DEB‐TACE	
**Volume (mL)**	281 (48.7‐1580)	.13	258 (45.0‐1301)	.15	−13% (−56% to 77%)	.39
**Volume/body weight (mL/kg)**	30.0 (5.70‐87.3)	**.03** ^*^	25.7 (3.90‐117)	**.02** ^*^		
**Ellipsoid tumor response classification**				Complete response	0 (0%)	N/A
				Partial response	3 (23%)	
				Stable disease	8 (62%)	
				Progressive disease	2 (15%)	
**Change in size**				Decreased	10 (77%)	.12
				Increased	3 (23%)	

Abbreviations: DEB‐TACE, drug‐eluting bead transarterial chemoembolization; HC, hepatic carcinoma.

^*^ *P* < .05.

All 16 dogs were discharged from the hospital 1 to 2 days after each DEB‐TACE treatment.

### 3.2 Complications

Eight out of 27 (30%) DEB‐TACE treatments resulted in mild complications^31^ in 6 individual dogs. Seven out of 27 (26%) treatments resulted in 5 dogs displaying signs of gastrointestinal disease <14 days after DEB‐TACE consistent with postembolization syndrome (PES), considered to be a mild complication.^31^ Signs of PES included vomiting (3/27; 11%), diarrhea (5/27; 19%), lethargy (1/27; 4%), and hyporexia (5/27; 19%). Postembolization syndrome signs occurred after both treatments in 2 dogs, after the first treatment in 1 dog and after the second treatment in 2 dogs. There was no association between the development of PES after the first treatment and the development of PES after the second treatment (*P* = 1.00). Embolization to stasis during either treatment was not associated with the development of PES (*P* = 1.00). A history of vomiting, diarrhea, or inappetence was not associated with the development of PES after DEB‐TACE (*P* = .47, .48, .4). There was no association between tumor size (mL or mL/kg) on pretreatment CT and the development of PES after the first treatment (*P* = 1.00, .53). There was no association between change in tumor size and the development of PES (*P* = .8).

Two dogs developed hemoabdomen 24 hours after initial DEB‐TACE; both required blood transfusions but not surgical intervention. None of the dogs with hemoabdomen reported before the DEB‐TACE developed hemoabdomen after DEB‐TACE. The development of a hemoabdomen after the first embolization was not associated with the development of PES after the first treatment (*P* = 1.00). Both dogs went on to receive a second DEB‐TACE treatment without development of hemoabdomen. The development of a hemoabdomen after initial DEB‐TACE was not associated with mass size on pre‐CT (volume, *P* = .49; volume/body weight *P* = .53).

Three out of 27 (11%) DEB‐TACE treatments resulted in major complications.^31^One dog became anorexic 16 days after DEB‐TACE and discharge from hospital. Bile peritonitis was diagnosed. The dog was euthanized due to gall bladder rupture suspected to be secondary to an acute infarct 3 weeks after DEB‐TACE. After the second treatment, 2 dogs of 11 dogs (18%) developed major complications.^31^ One dog presented 4 days after discharge with abdominal pain, vomiting, and fever. Abdominal exploratory revealed a septic abdomen with a nonresectable hepatic abscess and hepatic mass with severe hemorrhage. Euthanasia was elected. One dog developed a hepatic abscess several weeks after the second DEB‐TACE procedure. The methicillin‐resistant *Staphylococcus pseudintermedius* abscess eventually ruptured and the septic peritonitis was managed with oral and injectable antibiotics, surgical drainage, and multiple percutaneous drainages with resolution 8 months later. Ultimately, 2/16 dogs (13%) or 2/27 treatments (7%) resulted in death. Embolization to stasis in the first or second treatment was not associated with major complications (*P* = .23, *P* = 1.00). Two dogs that developed hepatic abscessation had tumor volumes (239 mL, 172 mL) and tumor volume/body weight (13.5 mL/kg, 8.43 mL/kg) smaller than the median values (285 mL, 30.0 mL/kg) for the study group. There was no association between a history of weight loss and complications after embolization (*P* = .3).

### 3.3 Long‐term outcomes

**FIGURE 1:**
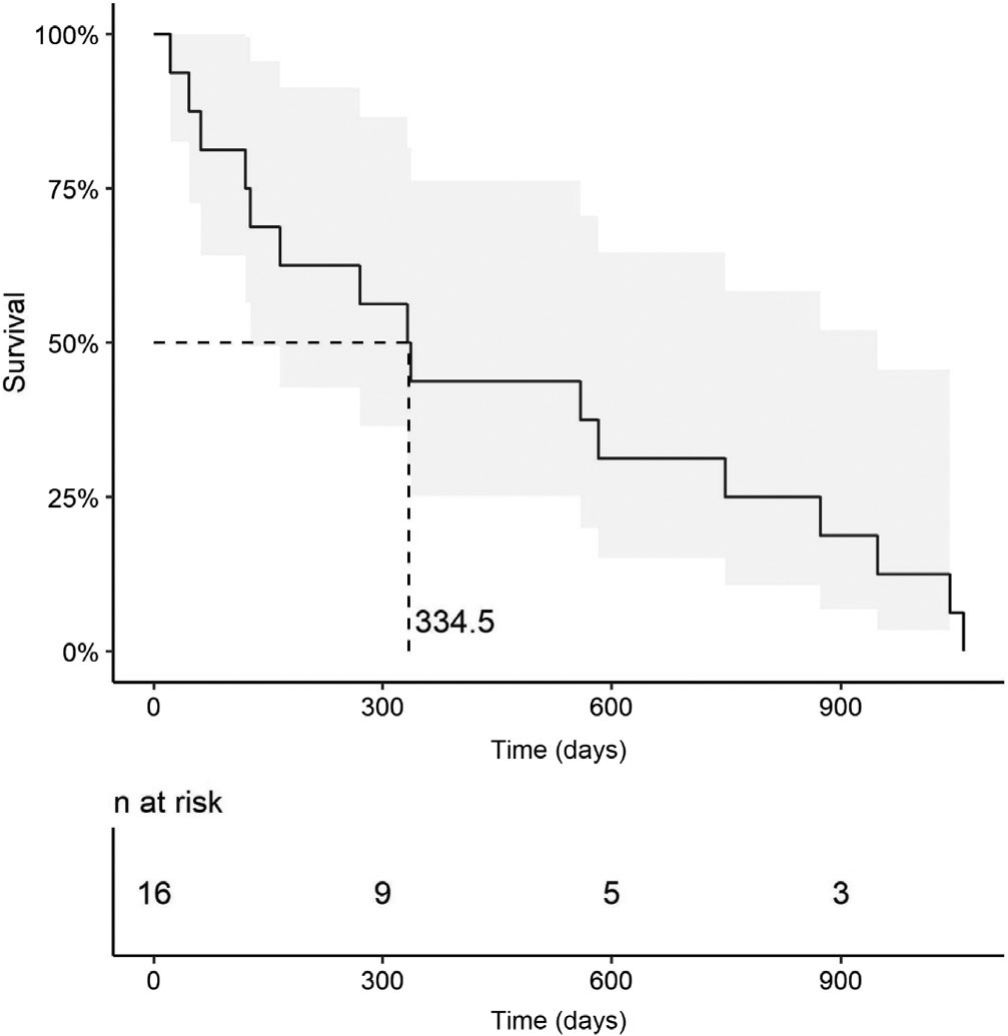
Kaplan‐Meier survival curve (solid line) and associated 95% confidence intervals for 16 dogs with HC treated with DEB‐TACE. DEB‐TACE, drug‐eluting bead transarterial chemoembolization; HC, hepatic carcinoma

**FIGURE 2:**
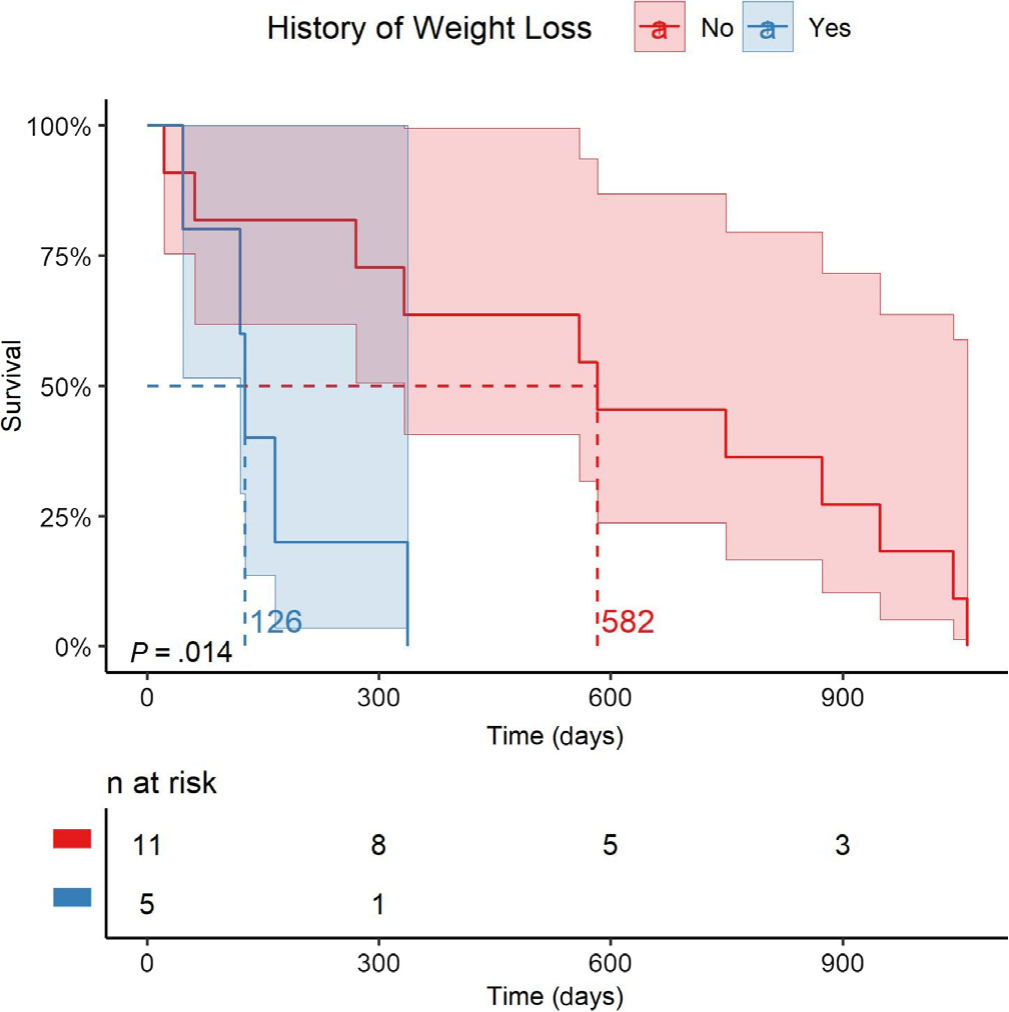
Kaplan‐Meier survival curves and associated 95% confidence intervals for 16 dogs with HC treated with DEB‐TACE with (blue) and without (red) a clinical history of weight loss. DEB‐TACE, drug‐eluting bead transarterial chemoembolization; HC, hepatic carcinoma

Median survival time for all of the dogs from the first DEB‐TACE treatment to date of death was 337 days (range, 22‐1061) (Kaplan‐Meier survival curve, Figure 1). A presenting complaint of weight loss was the only clinical/historical sign predictive of death (*P* = .02). Dogs with a history of weight loss had a significantly reduced ST of 126 days (range, 46‐337) compared to those dogs without a prior history of weight loss (582 days; range, 22‐1061) (Figure 2).

If the 2 dogs that died from treatment related complications were excluded from analysis, the MST for dogs without fatal procedural complications was 448 days (range, 61‐1061). Ten of 16 (63%) dogs were suspected to have died as a result of HC or DEB‐TACE. Having mild or severe complications after the first or second embolization was not associated with survival (*P* = .07, .99).

There was no association between PES, or specifically diarrhea, after the first embolization (*P* = .55, *P* = .99) or second embolization (*P* = .62, *P* = .95) and survival. Development of a hemoabdomen after DEB‐TACE was not associated with survival (*P* = .99). For the 2 dogs with a hemoabdomen after the procedure, 1 dog survived 582 days after‐DEB‐TACE and was ultimately euthanized due to an unknown reason, and the other dog was euthanized 337 days after DEB‐TACE because of a suspected hemoabdomen.

There was no association between the date of the first treatment and death as a result of the treatment, implying the absence of a substantial learning curve (*P* = .28).

There was no association between whether the first embolization was taken to stasis (*P* = .88) or whether there were complications after the first embolization (*P* = .62) and survival. There was no association between whether the second embolization was taken to stasis (*P* = .99) or whether there were complications after the second embolization (*P* = .6) and survival.

There was no association between tumor volume (mL) on pretreatment CT and survival (*P* = .13). There was a statistically significant association between larger tumor volume/body weight (mL/kg) on pretreatment CT and shorter survival (*P* = .03). There was no association between post‐treatment CT tumor volume (mL) and survival (*P* = .15); however, there was again a statistically significant association between post‐treatment CT larger tumor volume/body weight (mL/kg) and shorter survival (*P* = .02). CT tumor response criteria which were not significantly associated with survival included percent change in tumor volume, or percent change in tumor volume adjusted for bodyweight before or after treatment (*P* = .39, *P* = .39). There was no association between a history of weight loss and tumor size as volume (mL) or volume/body weight (mL/kg) (*P* = .44, *P* = .18).

### 3.4 Necropsy

Six out of 16 dogs (38%) had full (4/6; 67%) or partial (2/6; 33%) necropsy reports available; 2 out of 6 necropsy reports were limited in scope with the necropsy report of 1 dog was limited to the hepatic mass and the necropsy report of another dog was limited to the liver and gallbladder. Five out of 6 necropsy reports (83%) confirmed regrowth of HCC; the final necropsy report was consistent with HC. One dog had incompletely resected HCC with vascular invasion diagnosed via excisional biopsy 1608 days before DEB‐TACE with suspected HCC regrowth on CT; however, necropsy results were consistent with focal and metastatic HC, suspected to be of neuroendocrine origin. With suspected metastatic neuroendocrine carcinoma to the liver and lungs on necropsy, dog 13's necropsy report was the only necropsy report to describe distant metastasis. Two out of 5 (40%) necropsy reports describing the gallbladder reported cholecystitis. Both of these dogs had tumors adjacent to the gallbladder. Cholecystitis was mild in 1 dog and severe in another. Histopathology revealed thrombosis and DEBs (LC/DC Bead 100‐300 μm hydrogel microspheres, Biocompatibles UK Limited) within gallbladder vessels adjacent to the rupture suggestive of an acute infarct in 1 dog which who was euthanized due to gall bladder rupture. Five out of 6 (83%) necropsy reports described intratumoral DEBs present between a median of 530 days (range, 22‐1007 days) after the second treatment (LC/DC Bead 100‐300 μm hydrogel microspheres, Biocompatibles UK Limited). There was no association between the presence of cholangiocystitis (*P* = .62) or the presence of intralesional beads (LC/DC Bead 100‐300 μm hydrogel microspheres, Biocompatibles UK Limited) on necropsy and survival (*P* = .29).

## 4 DISCUSSION

There are currently no established therapeutic recommendations for dogs with metastatic, nonresectable, or incompletely excised HC.^42^ The results of this study showed that DEB‐TACE treatment for nonresectable HC could be performed successfully, resulted in few major complications, and resulted in 77% of tumors displaying a reduction in volume on follow‐up CTA. Drug‐eluting bead transarterial chemoembolization could emerge as a viable treatment alternative for dogs with nonresectable HC.

In DEB‐TACE in people, the most common complications after embolization are minor and include PES, a syndrome mediated by an inflammatory response to the embolization/chemotherapeutic agent, manifesting in self‐limiting abdominal pain, nausea, fever, and fatigue within 14 days of treatment.^19^, ^43^, ^44^ In people, PES is associated with a 2‐fold increased risk of death.^43^ The current study failed to demonstrate an association between PES in dogs and reduced STs; however, the study population was small and the lack of significance could be attributed to a type II statistical error. In people, diarrhea is a less common complication after DEB‐TACE, occurring about in ~1.6% of humans.^45^ Conversely, 5/27 (19%) of DEB‐TACE treatments resulted in transient diarrhea in the dogs in this study; prophylactic treatment should be considered.

The complications of hepatic abscess and cholecystitis occurred after 3/27 (11%) of treatments in dogs in this study. Embolization of the cystic artery, chemotherapeutic injury to bile ducts, deposition of lipiodol into the gallbladder wall, bacterial translocation from a necrotic embolized tumor, and the local immunosuppression from chemotherapeutics might contribute to these complications.^44^, ^46^, ^47^ In human medicine, the addition of local high‐dose antibiotics with embolics might reduce hepatic abscessation and could be investigated in dogs.^46^ Larger tumors and increased quantities of embolics are linked to hepatic abscessation in people.^44^ The relatively larger tumors treated in dogs might contribute to the higher risk of subsequent abscessation.

Hemoabdomen after TACE is a rare complication with in people with a risk of 0.2% to 0.4% per procedure and more common in larger, heterogeneous, subcapsular, exophytic tumors; these are common characteristics of nonresectable HC in dogs.^48^, ^49^, ^50^ In this study, hemoabdomen developed after 2 treatments (2/27; 7%). They were treated with packed red blood cell transfusions and survived to discharge for 337 days and 582 days, respectively. Postoperative hemoabdomen did not affect long‐term survival; however, this study had a small sample size, and a type II statistical error is possible. Subjectively, HCC in dogs tends to be larger and more cavitated compared to HCC in people, potentially increasing the risk of tumor rupture.

While this study showed no survival benefit of embolization to blood flow stasis, complications were comparable between both groups suggesting that more aggressive embolizations might be safe to perform. The small population also increases the risk of a type II statistical error. There were some dogs in which repeat embolizations were anticipated to be more difficult if the prior treatment had been performed to stasis, suggesting that if repeat procedures are planned, embolization to blood flow stasis during the initial treatment might be avoided, or more time should be allowed to pass between subsequent treatments. However, this study demonstrates the technical feasibility of pursuing a second DEB‐TACE treatment in 2 dogs that were previously embolized to blood flow stasis.

Clinical presentation might help identify dogs most likely to benefit from DEB‐TACE. Dogs presenting with weight loss before TACE‐DEB were identified to have a shorter MST in this study; 126 days MST if prior history of weight loss vs 582 day MST without prior history of weight loss. This could be due to a more biologically malignant behavior of tumors resulting in systemic weight loss, or comorbidities which were not completely captured by any of the other parameters evaluated.

Limitations of this study included variability in treatment protocols, absence of reference or negative control groups, small sample size, lack of additional follow‐up imaging, and the absence of modified response evaluation criteria in solid tumors and tumor perfusion to assess CT data. The small sample size allows for the high risk of a type II statistical error, so nonsignificant results should be interpreted with caution. Future studies comparing DEB‐TACE alone and in combination with other treatment modalities should be considered.

### 4.1 Clinical relevance

This study demonstrated the feasibility and safety of performing DEB‐TACE for nonresectable HC which promoted partial response in 23% of dogs, short‐term SD or partial response in 85% of dogs, and 77% of the tumor volumes reducing in size after treatment. These early results justify additional controlled clinical trials comparing DEB‐TACE to other treatment modalities. A significant relationship between pre‐DEB‐TACE and post‐DEB‐TACE CT tumor volume divided by body weight was found to significantly predict survival. Dogs with a smaller tumor burden (relative to body weight) survived longer. Additionally, dogs with a presenting complaint of weight loss had a significantly shorter MST than those dogs without prior history of weight loss.

## CONFLICT OF INTEREST DECLARATION

Dr Weisse and Dr Berent are consultants for Infiniti Medical, LLC. In addition, Dr Weisse is a minority equity holder of this same company. A Nord Grant provided financial support this work. Biocompatibles provided the DEBs for this study. None of the remaining authors had any personal or financial relationships that could inappropriately influence or bias the contents of the paper.

## OFF‐LABEL ANTIMICROBIAL DECLARATION

Authors declare no off‐label use of antimicrobials.

## INSTITUTIONAL ANIMAL CARE AND USE COMMITTEE (IACUC) OR OTHER APPROVAL DECLARATION

Approved by The Animal Medical Center. Each client provided consent and was counseled for participation in the study for each animal.

## HUMAN ETHICS APPROVAL DECLARATION

Authors declare human ethics approval was not needed for this study.
